# Leveraging PEPFAR for HIV drug equity

**DOI:** 10.3389/frph.2023.1105645

**Published:** 2023-04-21

**Authors:** Alessandro Hammond, Belson Rugwizangoga, Fatima Cody Stanford

**Affiliations:** ^1^ Harvard University, Cambridge, MA, United States; ^2^ Division of Hematology/Oncology, Department of Pediatric Oncology, Boston Children's Hospital, Boston, MA, United States; ^3^ Department of Pathology, School of Medicine and Pharmacy, University of Rwanda, Kigali, Rwanda; ^4^ Directorate of Research and Department of Pathology, University Teaching Hospital of Kigali, Kigali, Rwanda; ^5^ Massachusetts General Hospital, MGH Weight Center, Department of Medicine-Division of Endocrinology-Neuroendocrine, Department of Pediatrics-Division of Endocrinology, Nutrition Obesity Research Center at Harvard (NORCH), Harvard Medical School, Boston, MA, United States

**Keywords:** HIV, health equity, medicine, PEPFAR, HIV prevention

## Introduction

The only manufacturing company of cabotegravir, ViiV Healthcare, has potentially altered the state of HIV treatment in the developing world by agreeing to voluntarily license the drug cabotegravir for generic production and distribution in all LICs and lower-middle LICs (1). Access advocates had been calling on ViiV Healthcare, which began selling the drug in the European Union in December 2020, to license the drug to generic manufacturers voluntarily. Yet, as of October 2022, only Zimbabwe has approved the drug for sale, with most countries citing the cost of the drug as the reason not to move forward with approval (2). The necessity for more equitable pricing is apparent, as the countries hardest hit by HIV in sub-Saharan Africa face a long wait to get the drug (3). ViiV has set the cost of treatment at 22,200 USD annually, but prices higher than 100 USD annually for generic oral PrEP are too high for many low and middle-income countries (LMICs) (2, 3).

## Discussion

The new HIV medication, cabotegravir, has shown increased effectiveness at preventing infection than the standard method of pre-exposure prophylaxis (PrEP) (3). In particular, cabotegravir has shown increased effectiveness over oral daily tenofovir diphosphate plus emtricitabine (TDF-FTC) for HIV prevention in women (3). Experience with oral PrEP shows that many people find it challenging to take a pill daily, while others are reluctant because of HIV-related stigma (4). Taking long-acting injectable cabotegravir every 2 months can help overcome many of the objections users may have (5). Another issue with oral PrEP has been that even though demonstration projects were funded and started in the years following proof of efficacy, little coordination among funding and implementing agencies dragged out the incorporation of the drugs into high-quality programs (4). These insights from oral PrEP programs offers essential lessons for the delivery of cabotegravir for PrEP.

Despite the many potential benefits of implementing cabotegravir for PrEP, ViiV has been, according to some critics, slow to secure deals with generic manufacturers (6, 7). Calls to celebrate a changing tide in the pharmaceutical game are thus premature (1). Generics are still 4–5 years away, and until then, ViiV Healthcare will likely be the sole manufacturer of cabotegravir (1). There are other obstacles to providing the drug. HIV may develop resistance to cabotegravir in people already infected with HIV, and this resistance hampers current HIV treatments (2). Thus, time-consuming, and costly sensitive HIV tests are administered in the United States before approving the drug for each patient (2). International funding will be vital in making such tests available if regulators determine that these tests are needed. Furthermore, to avoid the issues with the global rollout of oral PrEP, demonstration projects need to be big and fast-acting to overcome people's hesitancy to ask for the drug (2). Central to combating stigma are increasing awareness, addressing misinformation or mistrust, and promoting effective use. Users also prefer to access PrEP in non-clinical settings, yet cabotegravir is injected in the buttocks, requiring clinical privacy (4). However, it is possible to have community-based drop-in centers offering comprehensive HIV prevention services (4). For cabotegravir to be game-changing, funds must be allocated to these supporting tasks. African LICs face increasing demand from their citizens to access HIV medication to accommodate the surges of cases (3). A stable population must require sustained ongoing use for a high-quality program to incorporate cabotegravir.

## Conclusion

A major potential solution is to use the clout of the President's Emergency Plan for AIDS Relief (PEPFAR), other donors, and national ministries of health to negotiate a price and volume guarantee that ensures a sustainable supply until generics are registered and readily available in approximately 4–5 years. While the current infrastructure amassed through PEPFAR is being used for testing and treatment of HIV, PEPFAR has not made a real attempt to utilize its funds to acquire cabotegravir (6). In fact, in July 2022, PEPFAR's Scientific Advisory Board (SAB) began discussions to procure the drug as ViiV Healthcare has promised to drop the price in certain low-income countries (LICs) (6). PEPFAR funding, which has provided 3.8 billion dollars for HIV services, should be allocated for cabotegravir acquisition (8).

We urge other donors and national ministries of health to negotiate a price and volume guarantee that ensures a sustainable supply to existing programs (1). Since ViiV is committed to allowing cabotegravir to be replicated by generics manufacturers, PEPFAR can function as a stopgap until the existence of generics lowers the price. Currently, the World Health Organization (WHO) is attempting to find a way for PEPFAR to implement injectable cabotegravir (9). The solution lies in PEPFAR to combat HIV in LICs and help achieve HIV equity. African LICs would benefit from leveraging PEPFAR networks, resources, and facilities to acquire and distribute cabotegravir. LIC health ministries must work with PEPFAR and other donors to treat HIV with cabotegravir and use the funds accessible to PEPFAR to acquire cabotegravir to allow for future HIV independence in LICs.
